# Spatial datasets of 30-year (1991–2020) average monthly total precipitation and minimum/maximum temperature for Canada and the United States

**DOI:** 10.1016/j.dib.2024.110561

**Published:** 2024-05-28

**Authors:** Heather MacDonald, Daniel W. McKenney, John Pedlar, Kevin Lawrence, Kaitlin de Boer, Michael F. Hutchinson

**Affiliations:** a Great Lakes Forestry Centre, Canadian Forest Service, Natural Resources Canada, P6A 2E5 1219 Queen Street East, Sault Ste. Marie, Ontario, Canada; b Fenner School of Environment and Society, Australian National University, Australia

**Keywords:** Grids, Raster, Temperature, Spatial datasets, Precipitation, Historical, Spline

## Abstract

Thin plate smoothing spline models, covering Canada and the continental United States, were developed using ANUSPLIN for 30-year (1991–2020) monthly mean maximum and minimum temperature and precipitation. These models employed monthly weather station values from the North American dataset published by National Oceanic and Atmospheric Administration's (NOAA's) National Centers for Environmental Information (NCEI). Maximum temperature mean absolute errors (MAEs) ranged between 0.54 °C and 0.64 °C (approaching measurement error), while minimum temperature MAEs were slightly higher, varying from 0.87 °C to 1.0 °C. On average, thirty-year precipitation estimates were accurate to within approximately 10 % of total precipitation levels, ranging from 9.0 % in the summer to 12.2 % in the winter. Error rates were higher in Canada compared to estimates in the United States, consistent with a less dense station network in Canada relative to the United States. Precipitation estimates in Canada exhibited MAEs representing 14.7 % of mean total precipitation compared to 9.7 % in the United States. The datasets exhibited minimal bias overall; 0.004 °C for maximum temperature, 0.01 °C for minimum temperature, and 0.5 % for precipitation. Winter months showed a greater dry bias (0.8 % of total winter precipitation) compared to other seasons (-0.4 % of precipitation). These 30-year gridded datasets are available at ∼2 km resolution.

Specifications Table

**Table:** 

Subject	Earth and Planetary Sciences
Specific subject area	Spatial Datasets of 30-year Average Monthly Total Precipitation and Mean Minimum/Maximum Temperature for Canada and the United States, 1991–2020
Data format	Analysed
Type of data	2 km resolution grids
Data Collection	In-situ measurement of minimum/maximum temperature and precipitation in Canada and the United States by the following networks: Environment and Climate Change Canada (ECCC), the National Weather Service (NWS) and Federal Aviation Administration stations at airports, the NWS Cooperative Observer Network, USDA Snow Telemetry (SNOTEL) network, and the citizen science Community Collaborative Rain, Hail and Snow (CoCoRaHS) Network. The mean monthly values from 1991 to 2020 were calculated using North American monthly homogenized maximum and minimum temperature data set, “Northam version “j” [1,2]. NOAA provided these data via ftp
Data source location	Canada / United States
Data accessibility	https://open.canada.ca/data/en/dataset/acd4c9f2-0598-47e8-aa4d-0a8a0964ce55 Raw data – Monthly averages used in calculating the 1991–2020 values: https://osf.io/u8yda/

## 1 Value of the Data

• These gridded temperature and precipitation datasets are commonly used to append historical climate estimates to remote locations for diverse applications, such as streamflow [3], groundwater recharge [4], droughts [5], heatwaves [6], flooding [7], river thermal regimes [8,9], frost [10], ecology [11], and other analyses.
• These datasets can be used as an average or baseline to evaluate trends, climate events, and provide context for year-to-year variability in temperature and precipitation.
• These products cover a recent normal period, 1991–2020, against which previous long-term averages can be compared.
• The high levels of accuracy reported for these 30-year datasets approach measurement error in some cases and are more accurate than comparable annual models. This data description also provides a case study using a published dataset and output from ANUSPLIN thin-plate spline program [12].

## 2 Background

A climate “normal,” defined by the World Meteorological Organization (WMO) as an arithmetic mean for a fixed 30-yr period [13], is used as a long-term measure to evaluate trends, climate events, and provide context for year-to-year variability in meteorological conditions. For the work reported here, spatial interpolation of calculated thirty-year averages for 1991 to 2020 was carried out using thin plate smoothing splines via ANUSPLIN [12]. The annual climate station data from which the 30-year normals were calculated was previously used to generate annual spatial models of monthly average minimum/maximum temperature and total precipitation [14]. The methodology was informed by the WMO's guidelines for the calculation of 1991–2020 climate normals [15].

## 3 Data Description

We introduce spatial datasets covering Canada and the US for 1991–2020 historical monthly mean minimum/maximum temperature and total precipitation created using thin plate smoothing splines via ANUSPLIN [12]. Climate station data used in this modelling effort were obtained from the North American dataset “j” [1,2] published by National Oceanic and Atmospheric Administration's (NOAA's) National Centers for Environmental Information (NCEI). We describe these datasets and report on the accuracy and bias of the spatial datasets.

**Fig. 1:**
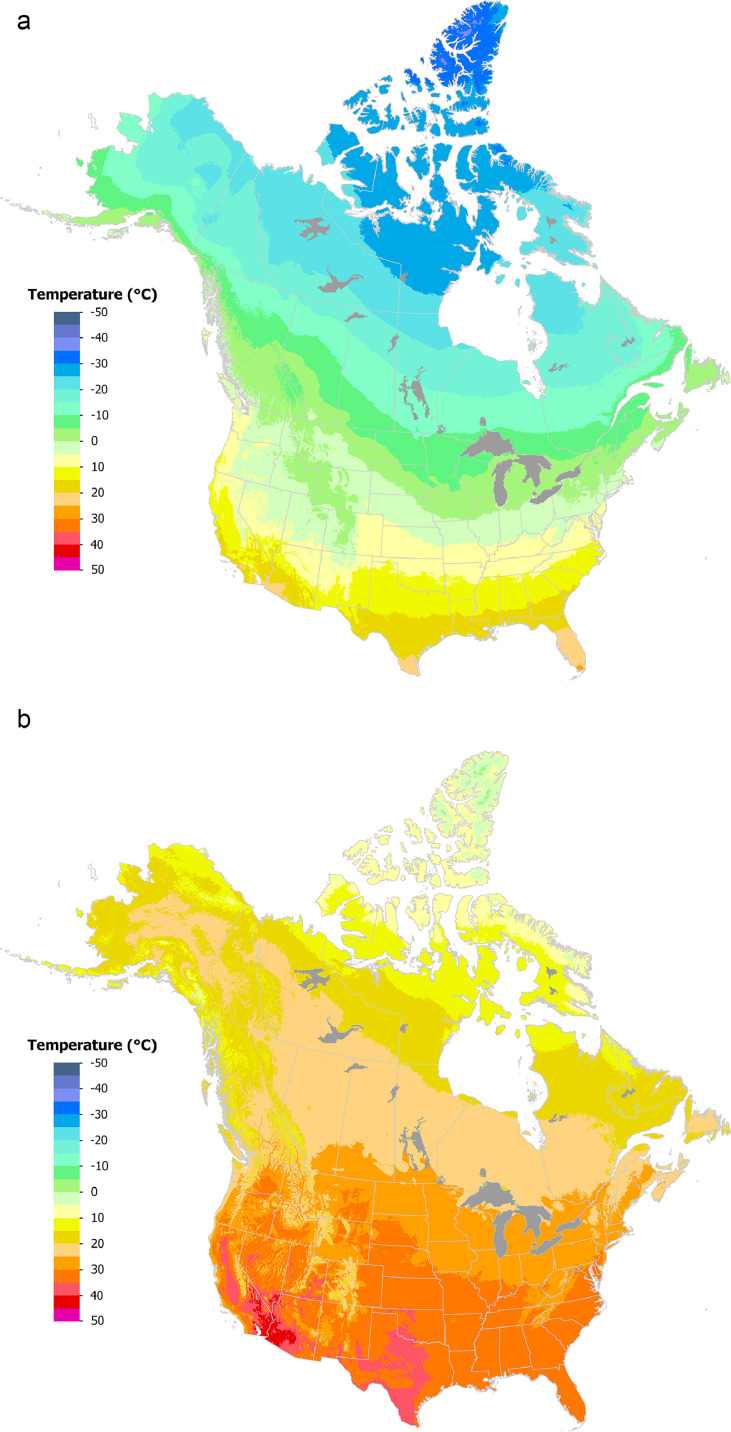

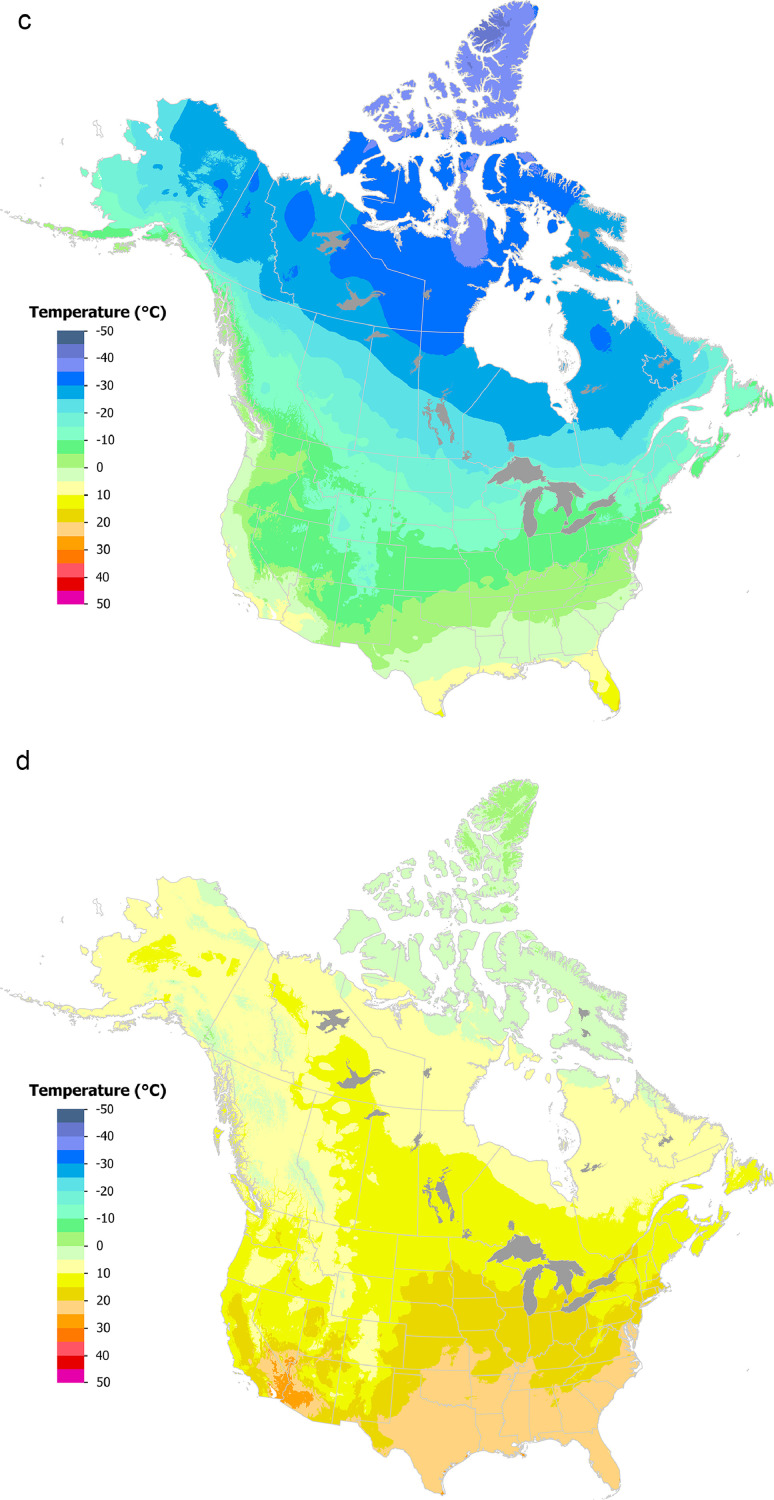

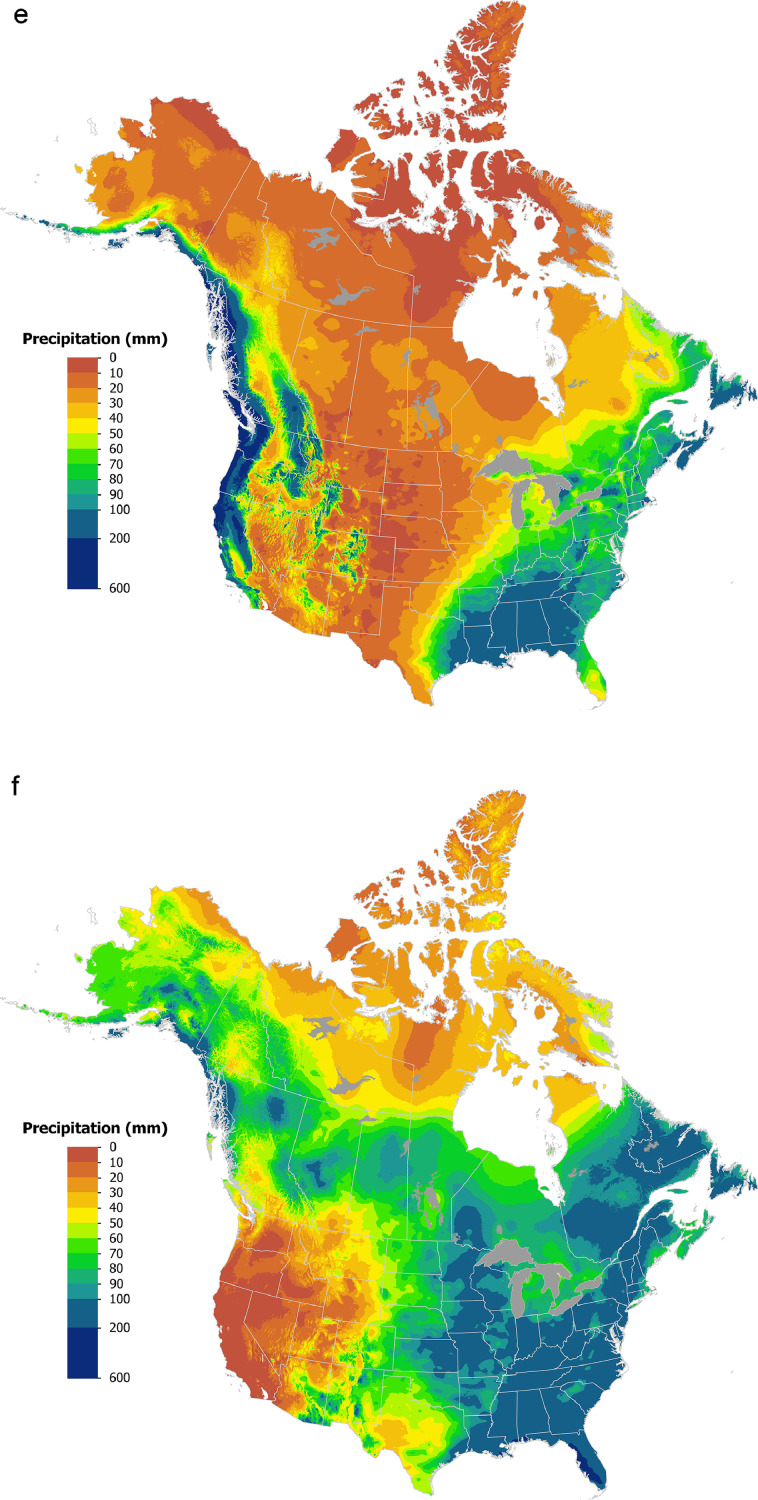
1991–2020 average maximum temperature (a, b); average minimum temperature (c, d); total precipitation (e, f) for January (a, c, e) and July (b, d, f). Temperature maps are presented in °C and precipitation maps are presented in mm.

Gridded datasets were generated covering Canada and the US for 1991–2020 monthly mean minimum/maximum temperature (Fig. 1a-d) and total monthly precipitation (Fig. 1e-f) using tri-variate thin-plate splines in ANUSPLIN [12] version 4.5 employing a 60” (approximately 2 km) Digital Elevation Model [16]: https://open.canada.ca/data/en/dataset/acd4c9f2-0598-47e8-aa4d-0a8a0964ce55

**Fig. 2:**
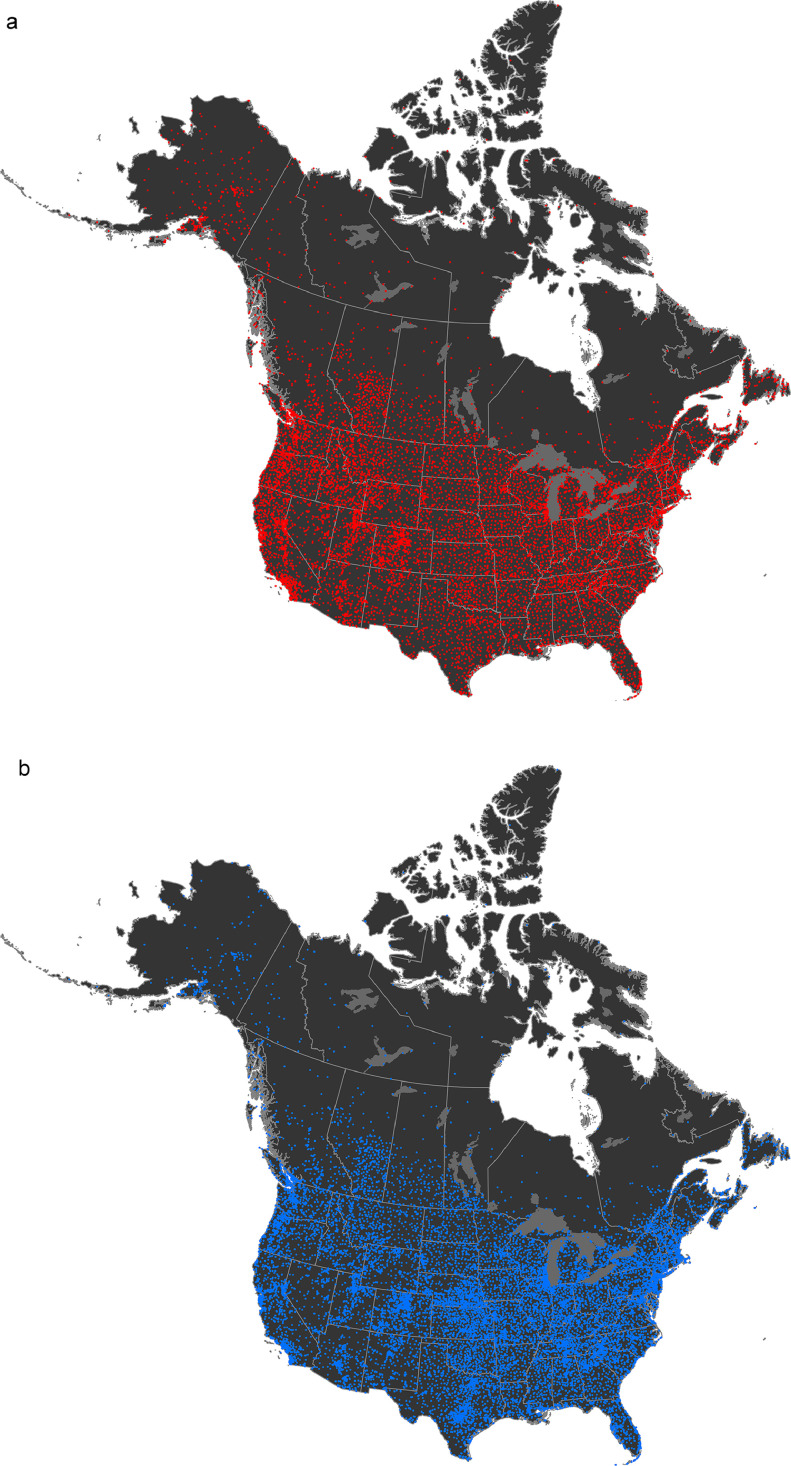
Map of Northam “j” temperature (a) and precipitation (b) stations used for the current models.

These datasets were developed based on 10,972 temperature and 16,194 precipitation stations in continental U.S. and Canada contained in the Northam “j” dataset (Fig. 2). The bulk of the stations were in the United States (8965 temperature and 14,201 precipitation stations) covering some 935.1 million hectares (including Alaska). In comparison, there were 2007 temperature stations and 2027 precipitation stations in Canada (Table 1) covering approximately 981.6 million hectares.

**Table 1:** Number of stations by years of station observation data by country over the 1991–2020 period.

Years of Data	TMAX			TMIN			PCP		
	CA	US	Total	CA	US	Total	CA	US	Total
10	105	297	402	107	286	393	107	758	865
11	104	203	307	121	243	364	124	888	1012
12	104	300	404	107	272	379	104	1045	1149
13	103	227	330	110	264	374	94	1049	1143
14	101	290	391	88	270	358	98	862	960
15	111	261	372	128	267	395	86	497	583
16	118	289	407	112	328	440	128	425	553
17	126	332	458	116	325	441	133	348	481
18	114	387	501	110	420	530	127	373	500
19	91	362	453	96	345	441	107	332	439
20	77	358	435	96	372	468	76	365	441
21	74	303	377	81	357	438	83	361	444
22	88	419	507	89	398	487	81	440	521
23	87	381	468	83	396	479	81	625	706
24	98	349	447	107	314	421	71	251	322
25	88	304	392	90	396	486	70	245	315
26	85	284	369	68	385	453	74	253	327
27	67	453	520	70	585	655	64	356	420
28	62	556	618	63	665	728	74	407	481
29	60	889	949	61	868	929	88	638	726
30	117	1766	1883	104	1209	1313	157	3700	3857
Total	**1980**	**9010**	**10,990**	**2007**	**8965**	**10,972**	**2027**	**14,218**	**16,245**

## 4 Experimental Design, Materials and Methods

### 4.1 Raw data

Average temperature and precipitation values for 1991 to 2020 were calculated using the North American monthly historical dataset (Northam version “j”) [1,2] published by the National Oceanic and Atmospheric Administration's (NOAA's) National Centers for Environmental Information (NCEI). Northam “j” is used by NOAA to develop U.S. Normals (Michael Palecki, personal comm.). Northam “j” is constructed using station observation data from Environment and Climate Change Canada (ECCC), the National Weather Service (NWS) and Federal Aviation Administration stations at airports, the NWS Cooperative Observer Network, USDA Snow Telemetry (SNOTEL) network, and the citizen science Community Collaborative Rain, Hail and Snow (CoCoRaHS) Network.

### 4.2 Data pre-processing

Following World Meteorological Organization (WMO) [17] guidelines, station-months with more than 11 missing days were first screened out of the analysis and then only stations with 10 or more years of actual values (rather than estimated) over the period of interest were included in the analysis. Quality control steps are detailed in T1 (see Data Availability), including comparisons to published U.S. temperature and precipitation normals for 1991–2020 [18,19].

### 4.3 Data processing

These spatial datasets were produced using ANUSPLIN [12], which, in this case, was configured to model climate variables as a trivariate spline function of latitude, longitude, and elevation. Like previous applications, approximately 40 % of data points were selected as knots [14,20]. Elevation scaling and precipitation transformations were the same as those used for developing monthly historical datasets [14]. The spatial models were resolved at 60 arc s or ∼2-km resolution using a North American digital elevation model [16].

### 4.4 Validation

Mean Absolute Error (MAE) and Mean Error (ME) were evaluated overall and by season: winter (December, January, February), spring (March, April, May), summer (June, July, August), and fall (September, October, November). These metrics make use of cross-validation errors provided by ANUSPLIN at each climate station (estimates generated with the station removed). For MAE, the CV estimates are converted to absolute values and then averaged (providing an overall measure of predictive accuracy), while for ME, the CV estimates are averaged directly (providing a measure of predictive bias). ME and MAE are presented in °C for temperature variables, and as a percentage of the monthly total for precipitation. MEs were compared for U.S. versus Canadian stations, partitioned by elevation (≤1000 m above sea level versus >1000 m above sea level).

A set of 160 stations was identified in previous work [14] as reflecting a geographically representative sample of high-quality, long-term stations across North America. We employed this same pool of stations in the current work; however, due to data quality criteria in the current study, the full sample of 160 stations was not available for all climate variables. To provide a sense of the spatial variation in model accuracy, predictive errors were mapped at this set of stations for January and July for maximum/minimum temperature as well as precipitation. Furthermore, this subset of high-quality stations was used to compare error rates for these thirty-year datasets to those for previously published annual monthly datasets [14] for the same period (1991 to 2020). Statistical testing using *t*-tests was implemented in SAS software, Version 9.4 of the SAS System for Windows.

#### 4.4.1 Mean absolute error (MAE)

*Temperature.* The 1991–2020 temperature variables were accurate on average within less than one degree Celsius (MAE: 0.59 °C – maximum temperature; 0.95 °C – minimum temperature, Table 2). Maximum temperature MAEs ranged between 0.54 °C and 0.64 °C (approaching measurement error) compared to minimum temperature MAES which varied between 0.87 °C and 1.0 °C. Higher minimum temperature errors reflect known challenges associated with factors such as cold air drainage [14].

**Table 2:** MAE (Mean Absolute Error) for 1991–2020 (CV estimates compared to recorded value) by Season for North America, Canada, and the United States.

Geography / Season	Maximum temperature ( °C)	Minimum temperature ( °C)	Precipitation (% of average total precipitation)
U.S. + Canada			
Winter (DJF)	0.58	0.97	12.2 %
Spring (MAM)	0.58	0.87	9.5 %
Summer (JJA)	0.64	0.96	9.0 %
Autumn (SON)	0.54	1.00	10.1 %
U.S. + Canada Total	0.59	0.95	10.2 %
Canada			
Winter (DJF)	0.46	0.89	18.1 %
Spring (MAM)	0.52	0.69	14.4 %
Summer (JJA)	0.59	0.73	10.6 %
Autumn (SON)	0.40	0.78	13.6 %
Canada Total	0.49	0.77	14.1 %
U.S.			
Winter (DJF)	0.61	0.98	11.5 %
Spring (MAM)	0.59	0.91	9.0 %
Summer (JJA)	0.67	1.01	8.8 %
Autumn (SON)	0.58	1.05	9.7 %
U.S. Total	0.61	0.99	9.7 %

*Precipitation.* Monthly mean precipitation MAEs were equivalent to 10.2 % of total precipitation on average, ranging from 9.0 % in the summer to 12.2 % in the winter. Precipitation estimates in Canada exhibited MAEs representing 14.7 % of mean total precipitation compared to 9.7 % in the United States (Table 2). MAEs were approximately twice as great at high elevation stations (>1000 m above sea level, Table 3).

**Table 3:** Precision of Prediction for ANUSPLIN datasets by elevation (<1000 m versus >= 1000 m), calculated as CV estimate less recorded.

Geography	Maximum temperature ( °C)		Minimum temperature ( °C)		Precipitation (% of average total precipitation)	
Season	<1000 m	>=1000m	<1000 m	>=1000m	<1000 m	>=1000m
U.S. + Canada						
Winter (DJF)	0.51	0.78	0.80	1.42	10.9 %	20.5 %
Spring (MAM)	0.55	0.67	0.75	1.21	8.5 %	16.3 %
Summer (JJA)	0.63	0.72	0.75	1.55	8.6 %	12.5 %
Autumn (SON)	0.51	0.66	0.83	1.49	9.4 %	15.9 %
U.S. + Canada Total	0.55	0.71	0.78	1.42	9.3 %	16.3 %
Canada						
Winter (DJF)	0.44	0.73	0.86	1.51	17.8 %	27.1 %
Spring (MAM)	0.52	0.49	0.67	0.95	14.1 %	23.2 %
Summer (JJA)	0.60	0.44	0.71	1.09	10.4 %	13.4 %
Autumn (SON)	0.39	0.49	0.76	1.15	13.3 %	23.0 %
Canada Total	0.49	0.54	0.75	1.18	13.9 %	21.7 %
U.S.						
Winter (DJF)	0.53	0.78	0.79	1.42	9.8 %	20.4 %
Spring (MAM)	0.56	0.67	0.78	1.22	7.8 %	16.1 %
Summer (JJA)	0.64	0.73	0.76	1.57	8.4 %	12.5 %
Autumn (SON)	0.54	0.67	0.85	1.50	8.8 %	15.7 %
U.S. Total	0.57	0.71	0.79	1.43	8.7 %	16.2 %

#### 4.4.2 Mean error (ME)

*Temperature.* MEs were less than 0.2 °C for maximum temperature and less than 0.3 °C for minimum temperature across analyses by season, country, and elevation (Tables 4 and 5). Overall, maximum temperature estimates were too warm on average by 0.004 °C whereas minimum temperature estimates were too cool on average by 0.01 °C (Table 4). High elevation stations (1000 m or more above sea level) showed greater bias. For high-elevation Canadian stations (i.e., fewer than 100 stations), ANUSPLIN estimates were too cool by 0.19 °C for winter maximum temperatures and by 0.21 °C for winter minimum temperature (Table 5).

**Table 4:** ME (Mean Error; CV estimate less recorded value) for 1991–2020 Average Minimum/Maximum Temperature and Precipitation for North America, Canada, and the United States.

Geography / Season	Maximum temperature ( °C)	Minimum temperature ( °C)	Precipitation (% of average total precipitation)
U.S. + Canada			
Winter (DJF)	0.003	−0.010	−0.8 %
Spring (MAM)	0.006	−0.006	−0.5 %
Summer (JJA)	0.004	−0.009	−0.2 %
Autumn (SON)	0.005	−0.012	−0.6 %
U.S. + Canada Total	0.004	−0.010	−0.5 %
Canada			
Winter (DJF)	0.000	−0.003	−1.5 %
Spring (MAM)	0.014	−0.006	−1.1 %
Summer (JJA)	0.017	−0.003	−0.3 %
Autumn (SON)	0.007	−0.010	−1.2 %
Canada Total	0.010	−0.005	−1.0 %
U.S.			
Winter (DJF)	0.004	−0.012	−0.7 %
Spring (MAM)	0.005	−0.006	−0.4 %
Summer (JJA)	0.001	−0.010	−0.2 %
Autumn (SON)	0.004	−0.013	−0.5 %
U.S. Total	0.003	−0.010	−0.4 %

**Table 5:** ME (Mean Error; CV estimate less recorded value) for ANUSPLIN datasets by elevation (<1000 m versus >= 1000 m), calculated as CV estimate less recorded.

Geography	Maximum temperature ( °C)		Minimum temperature ( °C)		Precipitation (% of average total precipitation)	
Season	<1000 m	>=1000m	<1000 m	>=1000m	<1000 m	>=1000m
U.S. + Canada						
Winter (DJF)	0.00	0.00	−0.01	−0.02	−0.44	−0.78
Spring (MAM)	0.00	0.01	0.00	−0.02	−0.30	−0.53
Summer (JJA)	0.00	0.02	0.00	−0.03	−0.17	−0.24
Autumn (SON)	0.00	0.01	−0.01	−0.03	−0.42	−0.50
U.S. + Canada Total	0.002	0.01	−0.005	−0.02	−0.33	−0.51
Canada						
Winter (DJF)	0.01	−0.19	0.01	−0.21	−0.90	−2.37
Spring (MAM)	0.01	0.09	0.00	−0.05	−0.63	−1.19
Summer (JJA)	0.01	0.08	0.00	0.00	−0.18	−1.33
Autumn (SON)	0.01	−0.06	−0.01	−0.06	−0.76	−2.60
Canada Total	0.01	−0.02	−0.001	−0.08	−0.62	−1.87
U.S.						
Winter (DJF)	0.00	0.01	−0.01	−0.01	−0.37	−0.73
Spring (MAM)	0.00	0.01	0.00	−0.02	−0.24	−0.51
Summer (JJA)	0.00	0.02	0.00	−0.03	−0.17	−0.20
Autumn (SON)	0.00	0.01	−0.01	−0.03	−0.36	−0.43
U.S. Total	0.001	0.01	−0.006	−0.02	−0.29	−0.47

*Precipitation.* Precipitation estimates were too dry by 0.5 % of the average monthly precipitation total (Table 4). Winter months showed a greater dry bias (by 0.8 % of total winter precipitation) compared to other seasons (dry bias of 0.4 % of precipitation). Precipitation estimates were more biased for Canadian locations (1.0 % dry bias compared to total precipitation) compared to U.S. locations (0.4 % dry bias). Precipitation estimates at locations above 1000 m above sea level were too dry by 0.51 % of total precipitation compared to 0.33 % at lower elevation locations. Particularly in the winter, stations at or above 1000 m above sea level exhibited greater bias (0.78 % too dry) compared to 0.44 % too dry for stations at elevations less than 1000 m above sea level (Table 5). For high elevation stations (>1000 m above sea level) in the U.S., ANUSPLIN precipitation estimates were 0.47 % too dry compared to 0.29 % too dry at lower elevation U.S. stations.

### 4.5 Mapping station errors

**Fig. 3:**
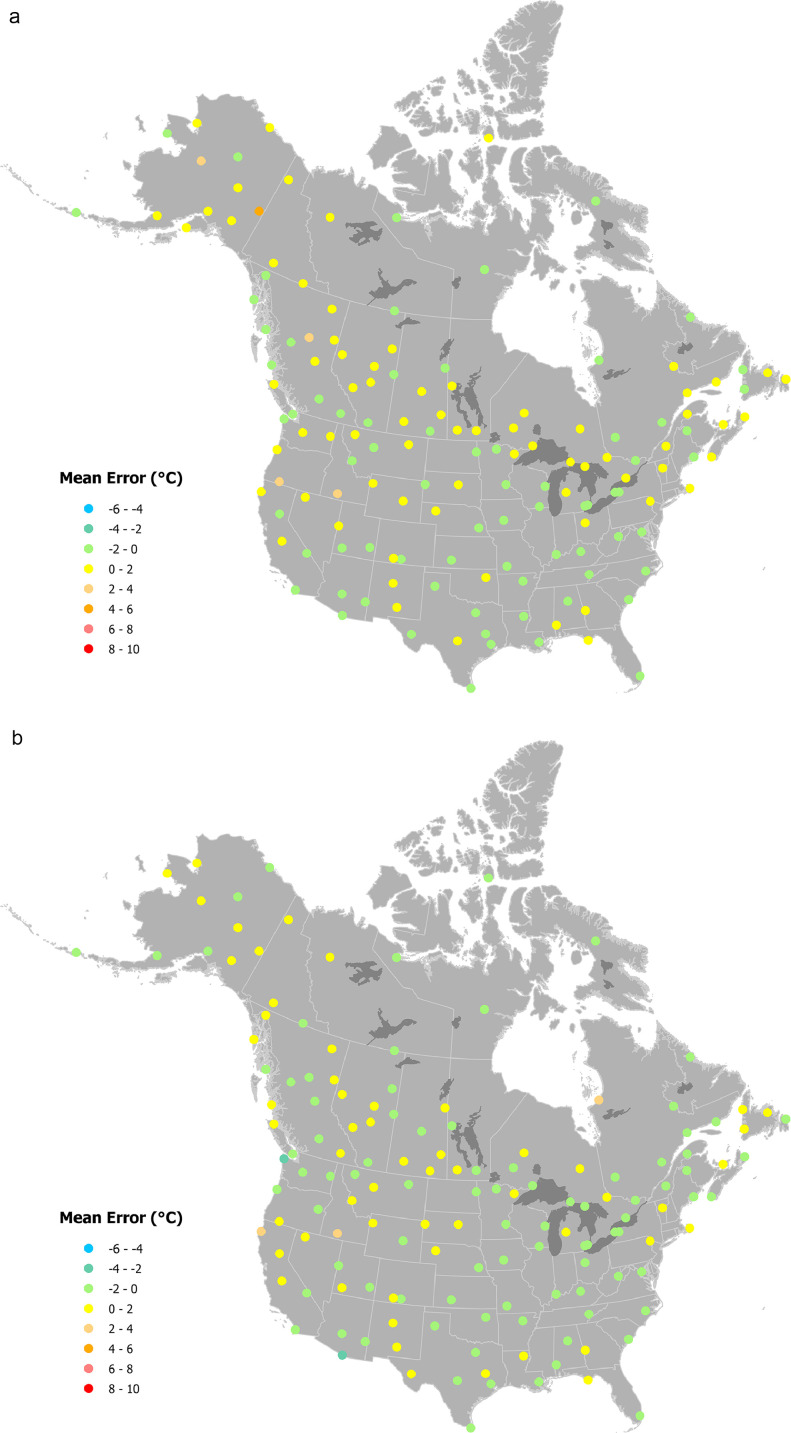
Spatial variation in errors at 160 weather stations for maximum temperature ( °C). Errors were calculated by subtracting actual recorded values from estimates for a) January and b) July.

**Fig. 4:**
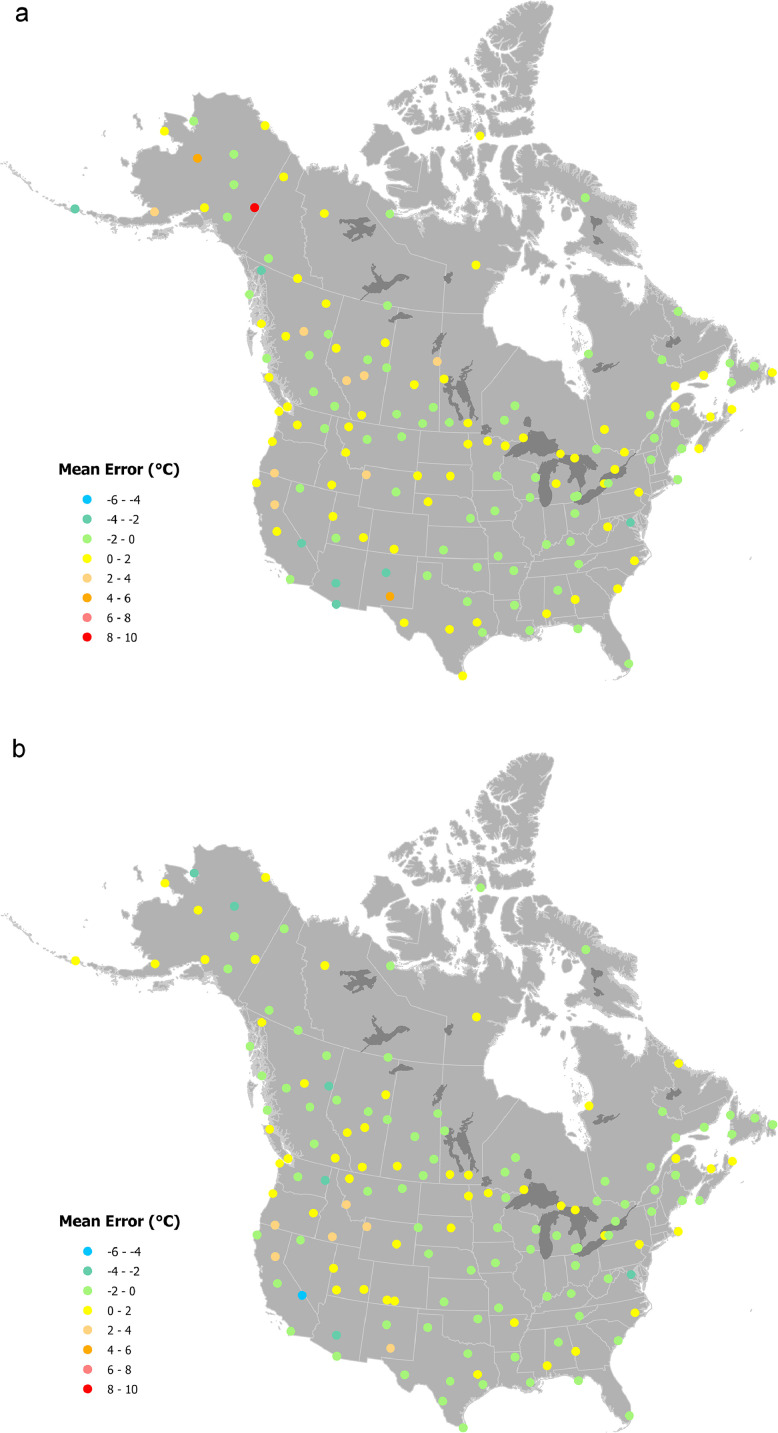
Spatial variation in errors at 160 weather stations for minimum temperature ( °C). Errors were calculated by subtracting actual recorded values from estimates for a) January and b) July.

Of the 160 stations included in the test sample, between 146 and 151 stations had sufficient data for error mapping, depending on the month and climate variable being mapped. Of the test stations, the greatest error for January maximum temperature was 4.61 °C (calculated as the ANUSPLIN estimate less the recorded value) for USC00501684 (64°05′30.1″N 141°55′15.6″W, Alaska), where the average maximum temperature was −24.21 °C compared to the ANUSPLIN estimate of −19.6 °C (Fig. 3a). Of the test stations, the largest absolute error for January minimum temperature was also at station USC00501684 (64°05′30.1″N 141°55′15.6″W, located in Alaska), which recorded minimum January temperature of −34.09 °C compared to the ANUSPLIN estimate of −25.99 °C, too warm by 8.1 °C (Fig. 4a). The next largest error for January minimum temperature was USC00503212 (64°44′26.9″N 156°52′33.6″W, also in Alaska) was much smaller at 4.4 °C (−27.2 °C recorded versus −22.8 °C estimated). The largest error for July minimum temperature was −4.37 °C for USC00042319 (36°27′43.9″N 116°52′01.2″W, in Death Valley California) in which recorded minimum July temperature was 32.82 °C and the ANUSPLIN estimate was 28.45 °C (Fig. 4b, CV less recorded).

**Fig. 5:**
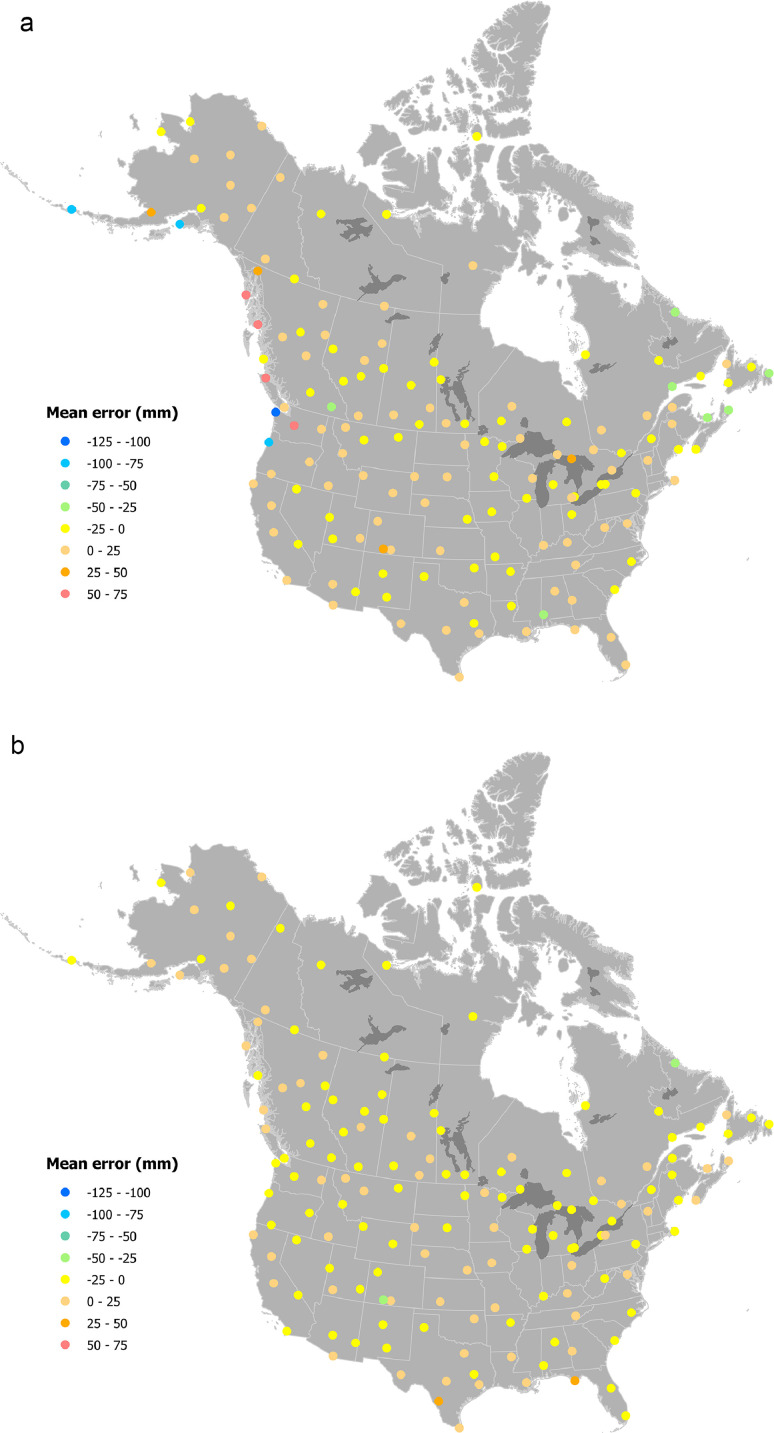
Spatial variation in errors at 160 weather stations for total precipitation (mm). Errors were calculated by subtracting actual recorded values from estimates for a) January and b) July.

The greatest underestimate for January total precipitation was Station USC00452914 (47°57′20.9″N 124°21′14.4″W, in Washington). The total recorded January precipitation for this station was 485.23 mm, compared to the ANUSPLIN estimate of 380.99 mm (Fig. 5a). In comparison, ANUSPLIN January precipitation estimate for Station CA001026270 (50°40′59.9″N 127°22′01.2″W, Port Hardy, British Columbia) was too wet by 68.83 mm (recorded precipitation of 238.22 mm compared to the ANUSPLIN estimate of 307.05 mm).

### 4.6 Comparison of monthly historical and 1991–2020 average ANUSPLIN datasets

The 1991–2020 thirty-year average datasets exhibited lower mean absolute error compared to the monthly historical datasets from 1991 to 2020, particularly for precipitation and minimum temperature estimates. The mean absolute errors for minimum temperature historical monthly estimates were 1.06 °C (January) and 0.9 °C (July) compared to 0.79 °C and 0.55 °C for the thirty-year average datasets (see T2 at the link provided in Data Availability). Maximum temperature absolute errors averaged 0.63 °C and 0.59 °C for January and July for the 30-year dataset compared to 0.72 °C and 0.68 °C for the monthly historical dataset. Precipitation MAEs for the 1991–2020 average dataset were 10.53 mm for January (2.3 % of the average precipitation monthly total) and 6.96 mm for July (2.5 %) compared to 14.69 mm and 18.17 mm respectively for annual monthly historical datasets from 1991 to 2020 (2.5 % and 3.9 % respectively).

## Limitations

The average historical total precipitation values used for the analysis were not adjusted for known measurement deficiencies [[21], [22], [23], [24]]. Initial testing (unpublished) of adjusted Canadian data merged with U.S. station data from NOAA showed discontinuity between U.S. and Canadian sides of the border. The precipitation grids described herein may therefore under-represent adjusted precipitation by 5 to 10 % in parts of southern Canada and by more than 20 % in parts of the Canadian Arctic compared to adjusted measures [21]. Future work may explore possible adjustments to recorded precipitation values near the border to produce a harmonized, adjusted precipitation gridded dataset for Canada and the United States.

Sparse distribution of in situ monitoring stations, particularly in Canada's north, is also a limitation of this dataset. In Canada, the number of stations at elevation of 1500 m or greater decreased from 71 in 1981–2010 to 9 stations in 1991–2020. In contrast, the number of high elevation stations in the United States increased from 778 stations in 1981–2010 to 1676 stations in 1991–2020. The documented decline in Canadian weather station density [25] reduces the accuracy of spatial interpolations. While there are some efforts to expand environmental monitoring, particularly in Canada's arctic areas [26], using Northam “j” for this study, the number of Canadian stations declined in the 1991–2020 period compared to the 1981–2010 period [14]. Future work will consider incorporating additional approaches for improving predictive ability [27]. On the other hand, bias estimates were low, averaging 0.01 °C too cool for minimum temperature, 0.004 °C too warm for maximum temperature, and 0.5 % too dry for temperature; all these errors are less than instrument precision [[28], [29]].

One shortcoming of global ANUSPLIN interpolations [30] is greater uncertainty in high elevation locations due to the sparsity of the monitoring network. ANUSPLIN interpolations at greater than 1000 m above sea level were less accurate compared to stations at lower elevations. For U.S. high elevation stations, temperature errors were relatively minimal; minimum temperature estimates were too cool by 0.02 °C, and maximum temperature estimates were too warm by 0.01 °C. While these error rates were higher than those of lower elevation U.S. stations (−0.006 °C and 0.001 °C respectively), estimates for U.S. high-elevation stations were relatively unbiased, related to the higher number of U.S. stations at elevations >1000 m above sea level (2600).

In contrast to the United States, there were fewer than 100 Canadian stations at >1000 m above sea level, which contributed to greater uncertainty in Canadian high elevation stations. The number of eligible available in situ Canadian stations has declined substantially between 1981 and 2010 and 1991–2020. While the number of U.S. stations eligible for calculation of our 30-year average increased between 1981 and 2010 and 1991–2020, the number of Canadian stations declined dramatically. The number of Canadian temperature stations in the 1991–2020 analysis dropped to 2007 from 3425 in 1981–2010, while the number of precipitation stations dropped to approximately 2000 from 3638 in 1981–2010. This level of monitoring in Canada is insufficient for what is “arguably the most fundamental attributes of the climate of a given locale” [19, p. 1687].

## Ethics Statement

This work did not involve human subjects or experiments using animals.

## CRediT author statement

**Heather MacDonald:** Conceptualization, Methodology, Formal Analysis, Validation, Writing -Original draft preparation, Writing - review & editing. **Daniel McKenney:** Conceptualization, Methodology, Writing –Original draft preparation, Writing - review & editing. **John Pedlar:** Conceptualization, Methodology, Visualization, Formal Analysis, Validation, Writing –Original draft preparation, Writing - review & editing. **Kevin Lawrence:** Data curation, Investigation, Validation. **Kaitlin de Boer:** Data curation, Investigation, Visualization, Investigation, Writing - review & editing. **Michael Hutchinson:** Software, Methodology, results validation and review.

## Data Availability

1991-2020 Average Monthly Total Precipitation and Minimum/Maximum Temperature for Canada and the United States (Original data).
